# Bridging the Gap: Integrating Pain into the Age-Friendly Health Systems 4Ms Framewor

**DOI:** 10.1093/geroni/igaf122.2186

**Published:** 2025-12-31

**Authors:** Beth Hogans, Raya Kheirbek

**Affiliations:** Johns Hopkins School of Medicine, Baltimore, Maryland, United States; University of Maryland School of Medicine, Baltimore, Maryland, United States

## Abstract

**Background:**

Pain is a prevalent yet often underrecognized factor in geriatric healthcare, significantly influencing quality of life, functional independence, medication use, and cognitive health. The 4Ms framework of Age-Friendly Health Systems (AFHS)—What Matters, Medication, Mentation, and Mobility—has redefined geriatric care by emphasizing patient-centered approaches. However, pain is not explicitly addressed within this model, despite its profound impact across all four domains. We hypothesize that pain has bidirectional relevance to each M and plays a crucial role in geriatric multicomplexity, the proposed ‘5th M’. Objective: This review explores the relationship between pain, the 4Ms, and geriatric multicomplexity, evaluating how pain influences each domain individually and collectively while assessing the potential consequences of its exclusion from AFHS frameworks.

**Methods:**

A comprehensive literature synthesis was conducted, analyzing peer-reviewed studies, clinical guidelines, and policy documents on pain, aging, and the 4Ms/5Ms models. Key focus areas included pain-associated conditions, barriers to effective management, pharmacological and non-pharmacological treatments, and cognitive and mobility impairments.

**Results:**

Pain profoundly affects patient priorities, increases polypharmacy risks, contributes to cognitive decline, and exacerbates functional limitations. It is both a consequence and a driver of multimorbidity, influencing healthcare utilization and social determinants of health.

**Conclusions:**

Integrating pain into AFHS models as a core component is essential for optimizing geriatric care. Recognizing its role across the 4Ms will lead to more effective healthcare policies, improved patient-centered treatment strategies, and enhanced quality of life for older adults. A policy shift toward routine pain assessment and multimodal management strategies is urgently needed.

